# Overall Survival in Phase 3 Clinical Trials and the Surveillance, Epidemiology, and End Results Database in Patients With Metastatic Colorectal Cancer, 1986-2016: A Systematic Review

**DOI:** 10.1001/jamanetworkopen.2022.13588

**Published:** 2022-05-24

**Authors:** Chan Shen, Daniel Tannenbaum, Robert Horn, Jane Rogers, Cathy Eng, Shouhao Zhou, Benny Johnson, Scott Kopetz, Van Morris, Michael Overman, Christine Parseghian, George J. Chang, Maria A. Lopez-Olivo, Raghav Kanwal, Lee M. Ellis, Arvind Dasari

**Affiliations:** 1 Division of Health Outcomes Research and Quality, Department of Surgery, Penn State College of Medicine, Hershey, Pennsylvania; 2 Division of Health Services and Behavioral Research, Department of Public Health Sciences, Penn State College of Medicine, Hershey, Pennsylvania; 3 Department of Internal Medicine, The University of Texas Health Sciences Center at Houston, Houston; 4 Department of Pharmacy Clinical Programs, the University of Texas MD Anderson Cancer Center, Houston; 5 Division of Hematology and Oncology, Vanderbilt-Ingram Cancer Center, Nashville, Tennessee; 6 Division of Biostatistics and Bioinformatics, Department of Public Health Sciences, Penn State College of Medicine, Hershey, Pennsylvania; 7 Department of Gastrointestinal Medical Oncology, The University of Texas MD Anderson Cancer Center, Houston; 8 Department of Colon and Rectal Surgery, The University of Texas MD Anderson Cancer Center, Houston; 9 Department of Health Services Research, The University of Texas MD Anderson Cancer Center, Houston

## Abstract

**Importance:**

Phase 3 trials for patients with metastatic colorectal cancer (mCRC) have been conducted with varying designs and often with surrogate end points for overall survival (OS).

**Objectives:**

To critically examine the factors associated with clinically relevant improvement in OS (defined as ≥2 months) in these trials and to evaluate their association with outcomes reflected in Surveillance, Epidemiology, and End Results (SEER) registry data.

**Evidence Review:**

Medline, EMBASE, Cochrane, Web of Science, ClinicalTrials.gov, EU Clinical Trials Register, and the International Clinical Trials Registry Platform were searched for phase 3 trials of systemic therapy for patients with mCRC by decade (1986-1996, 1997-2006, and 2007-2016), excluding early or pilot studies, studies that did not involve an anticancer drug, studies on cancer screening and prevention, reports of pooled data from multiple trials, and studies with nonpharmaceutical approaches. The association of drug development with OS outside the clinical trial setting was evaluated using data from the SEER registry, including adult patients with a primary cancer site in the colon or rectum, including adenocarcinoma, mucinous adenocarcinoma, or signet ring cell carcinoma; a distant stage; and receipt of chemotherapy as first-line therapy. Kaplan-Meier curves and log-rank tests were used to assess OS.

**Findings:**

The literature search identified 150 phase III clinical trials with 77 494 total enrollments, and 67 126 patients with mCRC were identified from the SEER database. Significant increases in survival were noted over time, best reflected in the experimental arm of first-line therapy (OS increased by 5.7 months per 10 years; 95% CI, 4.7-6.6 months; progression-free survival increased by 1.4 months per 10 years; 95% CI, 0.7-2.1 months). Although 69 of 148 trials (46.6%) met their predefined primary end point (including 20 of 44 trials [45.5%] with OS as the primary end point), only 35 of 132 trials (26.5%) resulted in improvement in OS by 2 months or more (including 13 of 42 trials [31.0%] with OS as the primary end point). Multivariable logistic regression showed that third-line therapies or later (odds ratio, 0.57; 95% CI, 0.51-0.63) and funding by pharmaceutical companies (odds ratio, 0.57; 95% CI, 0.54-0.60) were less often associated with improvement in OS. Furthermore, there was a decrease in the novelty of targets and agents over time, with trials that evaluated regimens composed entirely of previously approved drugs for mCRC increasing from 28% to 50%. Data from the SEER database showed that median OS increased from 12 months (95% CI, 12-13 months) (1986-1996) to 21 months (95% CI, 21-22 months) (2007-2015) (*P* < .001), but the 5-year OS continued to be low at 12.2% in 2011.

**Conclusions and Relevance:**

In this systematic review, OS for patients with mCRC appeared to improve significantly in trials, translating into meaningful benefits outside the clinical trial setting; however, these advances, although significant cumulatively, are largely incremental individually. These data should be a call to aim for larger gains from future trials with novel drugs, building on the increasing understanding of the biology of mCRC and sophisticated translational research tools.

## Key Points

**Question:**

How have phase 3 clinical trials for systemic therapy for metastatic colorectal cancer changed during the past 3 decades (1986-1996, 1997-2006, and 2007-2016), and what is their association with survival outcomes reflected in Surveillance, Epidemiology, and End Results (SEER) registry data?

**Findings:**

In this systematic review of 150 phase 3 clinical trials, fewer than one-third of the trials resulted in improvement of overall survival by 2 months or more, with later lines of therapy and funding by pharmaceutical companies being less often associated with such improvements. Data from the SEER registry showed incremental advances in survival, but long-term outcomes continue to be limited.

**Meaning:**

Although the cumulative incremental advances are encouraging, trials should work for larger gains with novel agents and against novel targets.

## Teaser

This systematic review examines the trial characteristics and cumulative progress of phase 3 clinical trials of systemic therapy for metastatic colorectal cancer over the last 3 decades and assesses the associated outcomes reported in the Surveillance, Epidemiology and End Results (SEER) database.

## Introduction

Metastatic colorectal cancer (mCRC) therapy has undergone significant improvements from when fluorouracil was the only active systemic therapy. Advances in systemic therapy have almost exclusively been through randomized phase 3 trials, which have a key limitation of addressing a single hypothesis in the midst of constantly evolving management paradigms that concomitantly contribute to improvements in survival.^1,2^ Furthermore, these trials are typically large and conducted during long periods at great expense but often yield negative results or incremental benefit.^3,4^ Here, we review the evolving characteristics of phase 3 trials of systemic therapies for patients with mCRC and summarize the cumulative progress and association with outcomes reflected in registry data. We also identify potential problems and propose ideas to improve future trials.

## Methods

### Data Collection

We created 2 databases for this study. The first database was of phase 3 trials for patients with mCRC. We conducted a systematic review following the methods suggested by the Cochrane Collaboration and report our methods based on the Preferred Reporting Items for Systematic Reviews and Meta-analyses (PRISMA) reporting guideline.^5,6^ We also aimed to evaluate the association of drug development with outcomes of patients outside the clinical trial setting through a second database of data from the Surveillance, Epidemiology, and End Results (SEER) registry by the National Cancer Institute of mCRC cases diagnosed from 1986 to 2015. To report the methods used to build the patient-level database, we used the Strengthening the Reporting of Observational Studies in Epidemiology (STROBE) reporting guideline.^7^ The University of Texas MD Anderson Cancer Center Institutional Review Board exempted this study from review because all patient-level data had been deidentified.

### Systematic Review

#### Eligibility Criteria, Data Sources, and Search Strategies

We included phase 3 clinical trials of systemic therapy for patients with mCRC with enrollment from January 1, 1986, to December 31, 2016. Exclusion criteria included early or pilot studies, studies that did not involve an anticancer drug, studies on cancer screening and prevention, reports of pooled data from multiple randomized clinical trials (RCTs), and studies that involved nonpharmaceutical approaches. We searched 6 electronic databases—Medline, EMBASE, Cochrane, Web of Science, ClinicalTrials.gov, EU Clinical Trials Register, and the International Clinical Trials Registry Platform—on July 26, 2018. Databases were searched using the following keyword search terms: *colorectal cancer* and *1986-present* and *survival/mortality* OR *progression free/recurrence/etc.* and *Cochrane RCT filter* and *RCT term *OR* controlled trial term* OR *Phase III term*. Two authors (J.R. and A.D.) reviewed trials for exclusion; disagreement was resolved by an independent reviewer (M.A.L.-O.).

#### Data Collection

Two authors (D.T. and R.H.) collected data independently and entered them into the database. Data collected by one author were cross-checked by the other, and disagreements were resolved by an independent reviewer (A.D.). We collected the following information on the trial characteristics: enrollment start year, trial size (number of patients enrolled or planned), line of therapy (first, second, or third and beyond), primary end point (overall survival [OS]; progression-free survival [PFS], time to progression, failure-free survival, or treatment-free survival [all referred to as PFS henceforth]; response rate [RR]; or other), and type of investigational agent (cytotoxic, targeted, other, cytotoxic and targeted, or cytotoxic and other). Trials that evaluated maintenance therapy were considered first line. Collected trial design information included whether the trial was placebo-controlled, whether it was blinded, the funding source (pharmaceutical company, nonprofit organization, or pharmaceutical company and nonprofit organization), and the study region (North America, Europe, Asia, multiregional, or unknown or not reported). Additional analyses included improvement in survival for OS or PFS (defined as ≥2 months) in addition to any improvement in RR. The cutoff of 2-month survival benefit was chosen based on an analysis of solid tumor drug approvals from 2002 to 2014 by the US Food and Drug Administration that showed median gains of 2.1 months for OS and 2.4 months for PFS.^8^ This cutoff is in fact conservative even compared with the recommendations by the American Society of Clinical Oncology and was chosen to reflect the ground realities and challenges of drug development in this space.^9^ Information regarding the investigational regimens was also obtained, including mechanism of action (cytotoxic, targeted, or other, including novel nontargeted chemical compounds, hormonal compounds, immunotherapy, biomodulator, cytotoxic and targeted, and cytotoxic and other), their novelty based on prior approval (for CRC, another oncologic indication or without prior oncologic indication), and based on target (target with prior drug approval for CRC or not).

#### Outcome and Synthesis Methods

For the trial-level data, we categorized the trials into 3 periods by start of enrollment: 1986 to 1996, 1997 to 2006, and 2007 to 2016. We provided the descriptive statistics, including numbers and percentages. We compared the trial characteristics by period using χ^2^ tests for categorical variables. We plotted the OS and PFS of experimental and control arms of the trials by year of enrollment and provided the fitted linear regression lines to show the time trend for first- and second-line therapies. We did not fit linear regression lines for third-line and beyond therapies because of the small sample sizes. We also conducted a multivariable logistic regression for positive OS improvement of 2 months or more. The model considered line of therapy, type of agent investigated, period, whether the investigated therapy was compared with a placebo, primary end point, and funding source. Odds ratios (ORs), 95% CIs, and *P* values were provided.

### SEER Data

We used the SEER 18 registry custom data with additional treatment fields (November 2018 submission).^10,11^ The SEER registry database covers approximately 35% of the US population and is a well-accepted source of information on cancer incidence and survival.^10,11^ The database provides detailed information on patient demographic characteristics, tumor characteristics, and survival. We restricted the sample using the following criteria to allow for reasonable comparison to patients enrolled in clinical trials: (1) adults at least 18 years of age; (2) primary site of colon or rectum; (3) 1 of 3 histologic subtypes: adenocarcinoma, mucinous adenocarcinoma, or signet ring cell carcinoma, corresponding to *International Classification of Diseases for Oncology* (*ICD-O-3*) codes 8140/3, 8180/3, or 8490/3; (4) distant stage; and (5) receipt of chemotherapy as first line of therapy.

### Statistical Analysis

Because the SEER database does not provide outcomes according to line of therapy, we provided the 1-year, 2-year, 3-year, and 5-year survival rates by year of diagnosis. We also provided Kaplan-Meier curves and log-rank test results by 3 periods for year of diagnosis: 1986 to 1996, 1997 to 2006, and 2007 to 2015. All statistical tests were 2-sided, and results are considered statistically significant at *P* < .05. All statistical analyses were conducted from January to July 2021 with SAS software, version 9.4 (SAS Institute Inc).

## Results

### Trial-Level Analyses

#### Study Selection and Characteristics

**Figure 1.**
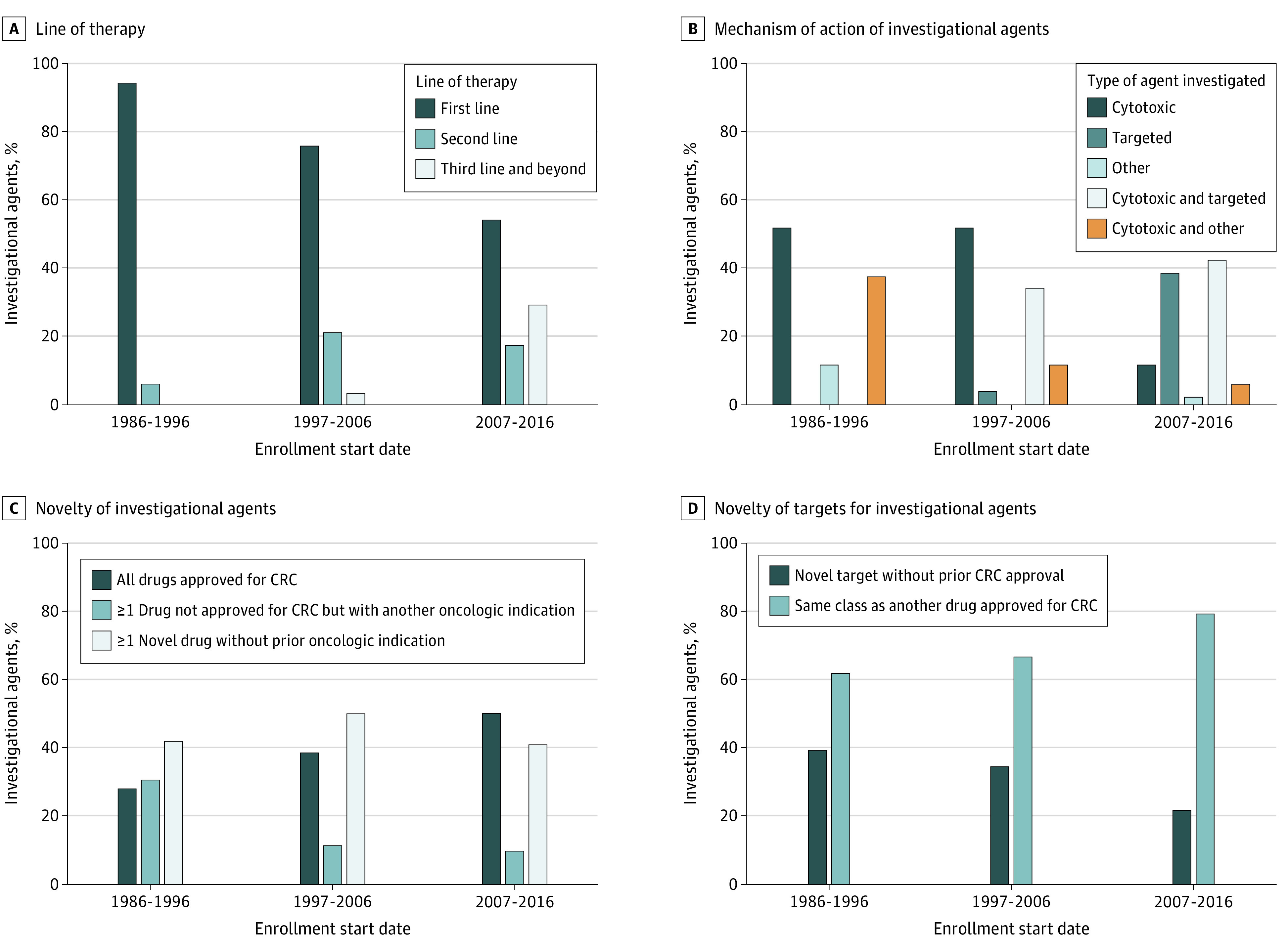
Characteristics of Investigational Agents Investigated in Phase 3 Colorectal Cancer (CRC) Clinical Trials

Of 3955 citations retrieved from our literature search, a total of 150 phase 3 clinical trials for mCRC with 77 494 total enrollments were included in this study (Table 1; eFigure and eAppendix in the Supplement). Study characteristics are given in Table 1; Figure 1 shows their evolution over time. First, these results show clear trends from 1986 to 1996 through 2007 to 2016 toward later lines of therapy, with first-line trials decreasing (from 34 of 36 [94.4%] to 28 of 52 [53.8%]) while second-line (from 2 of 36 [5.6%] to 9 of 52 [17.3%]) and third-line (from 0 to 15 of 52 [28.8%]) trials increased. Second, trials were more likely to be funded by pharmaceutical companies (pharmaceutical companies as sole funding source increased from 8 of 36 trials [22.2%] to 30 of 52 trials [58%], with those by nonprofit organization staying relatively stable at 11 of 36 [30.6%] vs 12 of 52 [23.1%]). Third, study regimens were more likely to include targeted agents alone (from 0 to 20 of 52 [38.5%]) or in combination with cytotoxic therapy (from 0 to 22 of 52 [42.3%]). However, a decrease was seen in the novelty of agents investigated, with trials of drugs against novel CRC targets (without prior approval) decreasing (from 14 of 36 [38.9%] to 11 of 52 [21.2%]) and those with all drugs already approved for CRC increasing (from 10 of 36 [27.8%] to 26 of 52 [50.0%]). Fourth, although the primary end point shifted toward PFS (from 5 of 36 [13.9%] to 23 of 52 [44.2%]) from overall response rate (from 13 of 36 [36.1%] to 1 of 52 [1.9%]), no significant change was found in the proportion of trials that met the per protocol predefined end point (15 of 36 [41.7%] vs 27 of 52 [54%]). Cumulatively, 40 271 patients were enrolled into negative trials. Fifth, drug development became more global, with Asia-only trials increasing from 0 to 12 of 52 trials (23.1%).

**Table 1.** Trial Characteristics and Outcomes by Study Period^a^

Characteristic or outcome	1986-1996 (n = 36)	1997-2006 (n = 62)	2007-2016 (n = 52)	*P* value
Trial characteristics				
Trial size, median (IQR), No. of participants	308 (198.5-430.5)	477.5 (291.0-820.0)	404 (248.0-756.0)	NA
Line of therapy				
First	34 (94.4)	47 (75.8)	28 (53.8)	<.001
Second	2 (5.6)	13 (21.0)	9 (17.3)	
Third and beyond	0	2 (3.2)	15 (28.8)	
Primary end point				
OS	14 (38.9)	16 (25.8)	16 (30.8)	<.001
PFS^b^	5 (13.9)	29 (46.8)	23 (44.2)	
Response rate	13 (36.1)	10 (16.1)	1 (1.9)	
Other^c^	4 (11.2)	7 (11.3)	12 (23.1)	
Treatment type				
Cytotoxic	18 (51.4)	32 (51.6)	6 (11.5)	<.001
Targeted	0	2 (3.2)	20 (38.5)	
Other	4 (11.4)	0	1 (1.9)	
Cytotoxic and targeted	0	21 (33.9)	22 (42.3)	
Cytotoxic and other	13 (37.1)	7 (11.3)	3 (5.8)	
Placebo				
Yes	3 (8.3)	8 (12.9)	18 (34.6)	.002
No	33 (91.7)	54 (87.1)	34 (65.4)	
Blinding				
Yes	4 (11.1)	7 (11.3)	19 (36.5)	.001
No	32 (88.9)	55 (88.7)	33 (63.5)	
Funding source				
Pharmaceutical company	8 (22.2)	27 (43.5)	30 (57.7)	<.001
Nonprofit organization	11 (30.6)	12 (19.4)	12 (23.1)	
Pharmaceutical company and nonprofit organization	17 (47.2)	23 (37.1)	10 (19.2)	
Sites				
North America	7 (19.4)	8 (12.9)	4 (7.7)	.001
Europe	19 (52.8)	30 (48.4)	16 (30.8)	
Asia	0	1 (1.6)	12 (23.1)	
Multiregion	10 (27.8)	21 (33.9)	18 (34.6)	
Unknown or not reported	0	2 (3.2)	2 (3.8)	
Trial outcomes				
OS				
No improvement	24 (72.7)	44 (77.2)	29/42 (69.0)	.66
At least 2 mo of improvement	9 (27.3)	13 (22.8)	13/42 (30.9)	
Positive primary end point				
Yes	15 (41.7)	27 (43.5)	27/50 (54.0)	.43
No	21 (58.3)	35 (56.5)	23/50 (46.0)	

Abbreviations: NA, not applicable; OS, overall survival; PFS, progression-free survival.

^a^ Data are presented as number (percentage) of patients unless otherwise indicated.

^b^ Progression-free survival includes failure-free survival, treatment-free survival, and time to progression.

^c^ Other end points included survival rate, toxic effects, and quality of life.

#### Changes in OS and Other Outcomes Over Time

**Figure 2.**
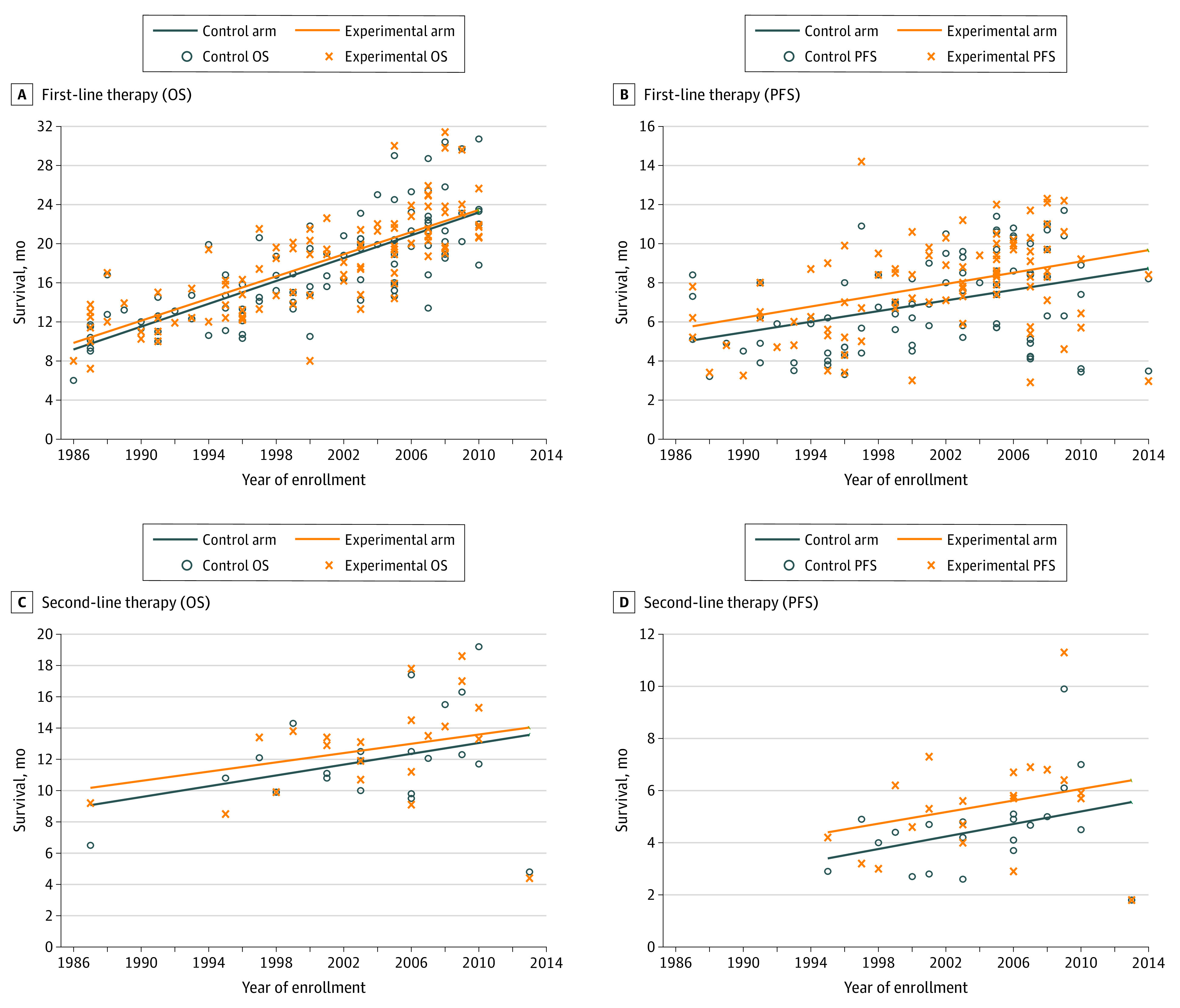
Time Trends for Outcomes in Phase 3 Colorectal Cancer Clinical Trials OS indicates overall survival; PFS, progression-free survival.

Figure 2 provides the OS and PFS of the experimental and control arms with linear regression lines to show the trend over time stratified by line of therapy when these outcome variables are available. Most of the trials were for first-line therapies (n = 109). Significant increases in survival were noted over time, best reflected in the experimental arm of first-line therapy (OS increased by 5.7 months per 10 years; 95% CI, 4.7-6.6 months; PFS increased by 1.4 months per 10 years; 95% CI, 0.7-2.1 months). Fewer second-line (n = 24) and third-line (n = 17) therapy trials had outcomes available, with others still ongoing without complete results at the time of data cutoff for this study. Nevertheless, these data did not show as clear an improvement in survival over time among second-line and especially third-line therapy trials.

#### Factors Associated With OS Improvement

Although 69 of 148 trials (46.6%) met their predefined primary end point (including 20 of 44 trials [45.5%] with OS as the primary end point, only 35 of 132 trials (26.5%) resulted in improvement in OS by 2 months or more (including 13 of 42 trials [31.0%] with OS as the primary end point. The multivariable logistic regression model for trial outcomes (Table 2) showed that second-line therapies were significantly more likely (OR, 1.31; 95% CI, 1.24-1.38; *P* < .001), whereas third-line therapies and beyond were significantly less likely (OR, 0.57; 95% CI, 0.51-0.63; *P* < .001) than first-line therapies to be associated with 2 months or more of OS improvement. Trials in the more recent years were much less likely to be associated with such improvement (OR, 0.39; 95% CI, 0.36-0.41 for 1997-2006 and OR, 0.42; 95% CI, 0.39-0.45 for 2007-2016; *P* < .001 for both) compared with 1986 to 1996. Trials funded by pharmaceutical companies were less likely to be associated with 2 months or more of OS improvement (OR, 0.57; 95% CI, 0.54-0.60; *P* < .001) than those funded by nonprofit organizations.

**Table 2.** Multivariable Logistic Regression for Positive Overall Survival Improvement by at Least 2 Months

Variable	OR (95% CI)	*P* value
Line of therapy		
Second	1.31 (1.24-1.38)	<.001
Third and beyond	0.57 (0.51-0.63)	<.001
First	1 [Reference]	NA
Treatment type		
Cytotoxic and other	0.15 (0.13-0.16)	<.001
Cytotoxic and targeted	1.45 (1.38-1.53)	<.001
Other	1.22 (1.07-1.40)	0.004
Targeted	3.08 (2.81-3.37)	<.001
Cytotoxic	1 [Reference]	NA
Study period		
1997-2006	0.39 (0.36-0.41)	<.001
2007-2016	0.42 (0.39-0.45)	<.001
1986-1996	1 [Reference]	NA
Placebo		
Yes	0.25 (0.24-0.27)	<.001
No	1 [Reference]	NA
Funding source		
Pharmaceutical company	0.57 (0.54-0.60)	<.001
Pharmaceutical company and nonprofit organization	0.57 (0.54-0.60)	<.001
Nonprofit organization	1 [Reference]	NA

Abbreviations: NA, not applicable OR, odds ratio.

### Patient-Level Analyses

**Figure 3.**
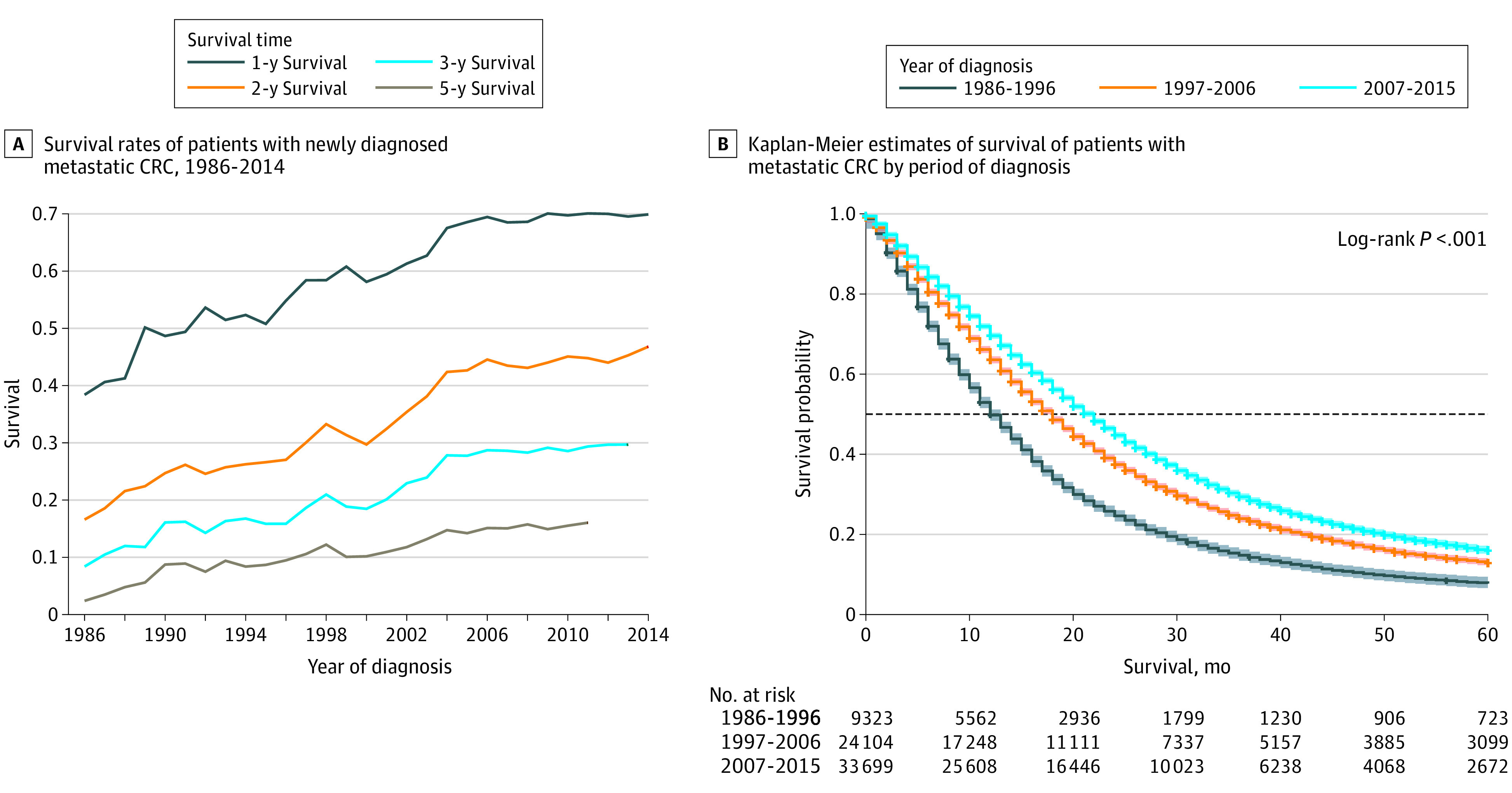
Survival Trends of Patients With Metastatic Colorectal Cancer (CRC) in the Surveillance, Epidemiology, and End Results Database A, Survival rates at 1, 2, 3, and 5 years for patients with metastatic CRC. B, Product limit survival estimates with 95% Hall-Wellner bands. Plus signs indicate censored data; horizontal line, 0.5 survival probability.

Using the SEER database, we identified 67 126 patients with mCRC who received chemotherapy from 1986 to 2015. Sample characteristics are given in the eTable in the Supplement. Figure 3A shows the 1-year, 2-year, 3-year, and 5-year survival rates during this time. We observed the most prominent improvements in the 1-year survival rate from less than 40% to approximately 70%, followed by 2-year survival from less than 20% to greater than 40%. Five-year survival also showed improvement, although it continued to be 12.16% in 2011. Figure 3B shows the Kaplan-Meier curves of OS by periods, providing clear evidence of the significant improvement in survival (log-rank *P* < .001). The median survival according to year of diagnosis improved from 12 months (95% CI, 12-13 months) in 1986 to 1996 to 21 months (95% CI, 21-22 months) in 2007 to 2015.

## Discussion

In this systematic review, we describe the methods, designs, and outcomes of mCRC systemic therapy trials conducted during the cytotoxic and targeted therapy eras (from 1986 to 2016) and evaluated secular trends of outcomes of patients with mCRC during the same period. Several important findings have emerged. A significant shift in the line of therapy in which trials are being conducted was observed from first to later lines, especially in the refractory setting. Parallel to this change, reflecting overall trends in oncology drug development, a significant shift toward the use of targeted therapy was also noted. During the same time, the proportion of pharmaceutical company–sponsored trials also increased significantly. Unfortunately, the proportion of trials that met the predefined per protocol primary end point has not changed significantly, with only a slight upward trend. Finally, trials that evaluated earlier lines of therapy appeared to provide more OS benefit compared with the refractory setting, with the advances in this setting only being incremental.

For the current study, a clinically significant improvement in survival was conservatively defined as 2 months or more of improvement over the control arm, which is much lower than the 4 to 5 months of survival benefit recommended by the American Society of Clinical Oncology Cancer Research Committee.^9^ Even then, although approximately half of the trials met their predefined end point, clinically meaningful benefit was noted in less than one-third of all trials, reflecting the prevailing chasm between statistically significant and clinically meaningful outcomes, a phenomenon noted in other tumor types as well.^12^ Evaluation of outcomes of patients with mCRC outside of clinical trials clearly shows that the 1- and 2-year survival rates have improved significantly. However, as previously reported,^13^ although advances in systemic therapy may have certainly contributed to the improved 1- and 2-year survival rates, they are also driven by improvement in diagnostic and surgical approaches, especially liver resection in patients with mCRC. The true effect of systemic therapy thus may be evaluated in long-term survival outcomes, which continue to be low.

An important lesson to be learned from the drug development of targeted agents is the need for well-designed biomarker trials: the approach adopted with the development of antiangiogenic agents in unselected patients has only yielded incremental benefits. This finding is in contrast to the recent successes noted with trials that used predefined biomarkers, such as high microsatellite instability, *BRAF* (OMIM 164757) V600E variant, *ERBB2* (OMIM 164870) (formerly *HER2* or *HER2/neu*) amplification, and *KRAS* (OMIM 190070) G12C variant.^14,15,16,17,18,19,20,21,22,23^ The common theme underlying the successes of all these trials is the bidirectional flow of data from the bench to the bedside. Future trials may also consider a similar approach by designing smaller biomarker-driven trials iteratively to fully capitalize the potential of precision oncology in mCRC.

Of interest, funding source appeared to be significantly associated with positive OS, with trials supported by solely nonprofit organizations having a higher chance of positive outcomes. Although this association is a topic of much debate, it has been noted in other tumor types as well. However, therapeutic advances, such as agents that target *RAS*, *ERRB*, *BRAF,* and high microsatellite instability protein in mCRC, would not be possible without a robust collaboration between industry and academia. Two key takeaways must be highlighted. First, the main driver for drug development by pharmaceutical companies is financial incentive; thus, decisions are driven as much by complex business factors as by science. These processes are for the most part outside the purview of clinicians, who can however use consensus definitions of clinical benefit (such as the one mentioned above by Ellis et al^9^) while designing collaborative trials with pharmaceutical companies. Second, certain questions that may not be financially attractive for pharmaceutical companies are still critical to be answered. For example, the Cancer and Leukemia B and Southwest Oncology Group 80405 trial could not have been conducted outside a cooperative group and yet helped answer many critical questions about optimal first-line mCRC chemotherapy regimens. In addition, this trial also yielded an immensely rich clinical database and biorepository that helped refine the use of epidermal growth factor receptor antibodies.

Prior studies^24,25,26,27^ have shown the challenges of conducting clinical trials in the US, including higher costs and underrepresentation of ethnic minorities. These findings are especially important given the underlying genetic differences among patient populations that may influence drug metabolism and/or efficacy. Acknowledging these trends and challenges, the US Food and Drug Administration has in fact issued guidelines toward acceptance of foreign clinical studies, including those not conducted under an Investigational New Drug application, which could accelerate drug development and approval.^28^ Thus, in the current study, as an exploratory objective, we aimed to evaluate the shifts in the location of the conduct of mCRC clinical trials. We found that significantly more trials were conducted solely in Asia over time (0% to 23%). Efforts toward reducing the costs of clinical trials through telehealth and other approaches for inclusion of minority groups in the US should also be made priorities for future studies.^29^

### Limitations

Our study is not without limitations. First, the study was limited to phase 3 clinical trials that evaluated systemic therapies in the metastatic setting within the defined search dates. Thus, the impact of advances outside these criteria (eg, phase 2 trials, improvements in surgery, supportive care, and advances from translational research) were excluded. Second, we were unable to completely account for confounding patient factors that may have accounted for improvements in survival, such as younger age, earlier diagnosis, and better performance status. However, our primary goal was to help with the study design of the next generation of phase 3 systemic therapeutic trials that have the most at stake. The SEER database only provides limited data regarding the systemic therapy delivered; however, as previously reported,^30^ increases in survival, especially beyond the middle of 2000, have been largely driven by improvements in systemic therapy coinciding with the drug approvals for the treatment of mCRC. Two additional key issues that have not been addressed in our analysis are adverse events and financial costs. Although negative clinical trial results cannot be completely avoided, they do lead to increased costs for drug development and increased toxic effects in patients. Furthermore, approved drugs are not always easily tolerable or cost-effective. These issues have been investigated extensively, and several frameworks exist that may be used during drug development.^31,32,33^ Third, our definition of a positive trial as the primary end point having a statistically significant difference in favor of the experimental arm may be too rigid and may not allow the more nuanced interpretation of results necessary when applying data clinically at the population level.^34,35^

## Conclusions

The results of this systematic review indicate that significant advances have been made in the median survival of patients with mCRC during the cytotoxic and targeted therapy eras. Although these advances are commendable, important lessons regarding trial design must be kept in mind to ensure clinically meaningful outcomes with an eventual goal of improvement in long-term OS of mCRC in the precision oncology era.

## Notes

Supplement. **eFigure.** PRISMA Flowchart (Adapted from "Moher D, Liberati A, Tetzlaff J, Altman DG,. PLoS Med 6(7): e1000097. doi:10.1371/journal.pmed1000097") **eTable.** Sample Characteristics of 67,126 mCRC Patients Who Received Chemotherapy From the SEER Database 1986-2015 **eAppendix.** Citations for Phase 3 Trials Included in the Study Arranged in Alphabetical Order of First Author Name
