# Supplementary material for: Overall Survival in Phase 3 Clinical Trials and the Surveillance, Epidemiology, and End Results Database in Patients With Metastatic Colorectal Cancer, 1986-2016: A Systematic Review
Source: JAMA Netw Open. 2022 May 24;5(5):e2213588. doi: 10.1001/jamanetworkopen.2022.13588 (PMC9131746; doi:10.1001/jamanetworkopen.2022.13588)
Supplement: Supplement. — eFigure. PRISMA Flowchart (Adapted from "Moher D, Liberati A, Tetzlaff J, Altman DG,. PLoS Med 6(7): e1000097. doi:10.1371/journal.pmed1000097") eTable. Sample Characteristics of 67,126 mCRC Patients Who Received Chemotherapy From the SEER Database 1986-2015 eAppendix. Citations for Phase 3 Trials Included in the Study Arranged in Alphabetical Order of First Author Name [file jamanetwopen-e2213588-s001.pdf]

Supplementary Online Content

Shen C, Tannenbaum D, Horn R, et al. Overall survival in phase 3 clinical trials and the Surveillance, Epidemiology, and End Results database in patients with metastatic colorectal cancer, 1986-2016: a systematic review. *JAMA Netw Open*. 2022;5(5):e2213588. doi:10.1001/jamanetworkopen.2022.13588

eFigure. PRISMA Flow Chart (Adapted from "Moher D, Liberati A, Tetzlaff J, Altman DG, PLoS Med 6(7): e1000097. doi:10.1371/journal.pmed1000097")

eTable. Sample Characteristics of 67,126 mCRC Patients Who Received Chemotherapy From the SEER database 1986-2015

eAppendix. Citations for Phase 3 Trials Included in the Study Arranged in Alphabetical Order of First Author Name

This supplementary material has been provided by the authors to give readers additional information about their work.

© 2022 Shen C et al. *JAMA Network Open.*

eFigure. PRISMA Flow Chart (Adapted from "Moher D, Liberati A, Tetzlaff J, Altman DG,. PLoS Med 6(7): e1000097. doi:10.1371/journal.pmed1000097")

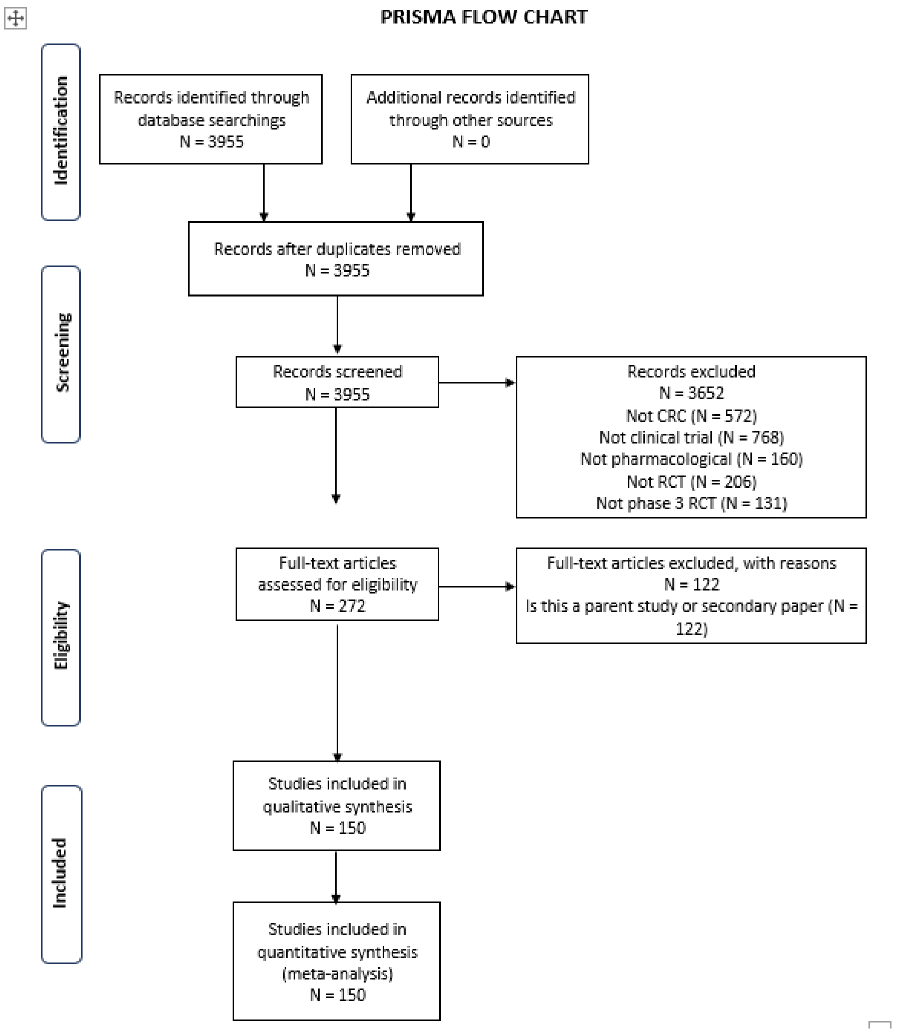

© 2022 Shen C et al. *JAMA Network Open.*

eTable. Sample Characteristics of 67,126 mCRC Patients Who Received Chemotherapy From the SEER database 1986-2015

	N (%)
**Age at diagnosis**	
Mean (SD)	61.8 (12.80)
Median	62.0
Interquartile range	53.0, 71.0
**Sex**	
Male	37402 (55.7%)
Female	29724 (44.3%)
**Race**	
Black	8549 (12.7%)
White	52735 (78.6%)
Other	5842 (8.7%)*
**Hispanic Origin**	
Non-Spanish-Hispanic-Latino	60590 (90.3%)
Spanish-Hispanic-Latino	6536 (9.7%)
**Marital Status**	
Single	10298 (15.3%)
Married	39391 (58.7%)
Other	17437 (26%)
**Histology**	
Adenocarcinoma, NOS	60031 (89.4%)

© 2022 Shen C et al. *JAMA Network Open.*

	N (%)
Mucinous adenocarcinoma	5582 (8.3%)
Signet ring cell carcinoma	1513 (2.3%)
**Year of diagnosis**	
1986-1996	9323 (13.9%)
1997-2006	24104 (35.9%)
2007-2015	33699 (50.2%)

*Other includes American Indian / Alaska Native, Asian, Pacific Islander and unknown

© 2022 Shen C et al. *JAMA Network Open.*

eAppendix. Citations for Phase 3 Trials Included in the Study Arranged in Alphabetical Order of First Author Name

1. Phase III randomized study of two fluorouracil combinations with either interferon alfa-2a or leucovorin for advanced colorectal cancer. Corfu-A Study Group. *Journal of clinical oncology : official journal of the American Society of Clinical Oncology*. 1995;13(4):921-928.

2. Oxaliplatin combinations as first-line therapy in advanced colorectal cancer. *Clinical colorectal cancer*. 2002;2(2):78-80.

3. Adams R, Brown E, Brown L, et al. Inhibition of EGFR, HER2, and HER3 signalling in patients with colorectal cancer wild-type for BRAF, PIK3CA, KRAS, and NRAS (FOCUS4-D): a phase 2-3 randomised trial. *The lancet Gastroenterology & hepatology*. 2018;3(3):162-171.

4. Adams RA, Meade AM, Seymour MT, et al. Intermittent versus continuous oxaliplatin and fluoropyrimidine combination chemotherapy for first-line treatment of advanced colorectal cancer: results of the randomised phase 3 MRC COIN trial. *The Lancet Oncology*. 2011;12(7):642-653.

5. Antonuzzo L, Giommoni E, Pastorelli D, et al. Bevacizumab plus XELOX as first-line treatment of metastatic colorectal cancer: The OBELIX study. *World journal of gastroenterology*. 2015;21(23):7281-7288.

6. Aparicio T, Ghiringhelli F, Boige V, et al. Bevacizumab Maintenance Versus No Maintenance During Chemotherapy-Free Intervals in Metastatic Colorectal Cancer: A Randomized Phase III Trial (PRODIGE 9). *Journal of clinical oncology : official journal of the American Society of Clinical Oncology*. 2018;36(7):674-681.

7. Aparicio T, Lavau-Denes S, Phelip JM, et al. Randomized phase III trial in elderly patients comparing LV5FU2 with or without irinotecan for first-line treatment of metastatic colorectal cancer (FFCD 2001-02). *Annals of oncology : official journal of the European Society for Medical Oncology*. 2016;27(1):121-127.

8. Aranda E, Díaz-Rubio E, Cervantes A, et al. Randomized trial comparing monthly low-dose leucovorin and fluorouracil bolus with weekly high-dose 48-hour continuous-infusion fluorouracil for advanced colorectal cancer: a Spanish Cooperative Group for Gastrointestinal Tumor Therapy (TTD) study. *Annals of oncology : official journal of the European Society for Medical Oncology*. 1998;9(7):727-731.

© 2022 Shen C et al. *JAMA Network Open.*

9. Avallone A, Piccirillo MC, Aloj L, et al. A randomized phase 3 study on the optimization of the combination of bevacizumab with FOLFOX/OXXEL in the treatment of patients with metastatic colorectal cancer-OBELICS (Optimization of BEvacizumab scheduLIng within Chemotherapy Scheme). *BMC cancer.* 2016;16:69.

10. Bandealy MT, Gonin R, Loehrer PJ, Monaco F, Einhorn LH. Prospective randomized trial of 5-fluorouracil versus 5-fluorouracil plus levamisole in the treatment of metastatic colorectal cancer: a Hoosier Oncology Group trial. *Clinical cancer research : an official journal of the American Association for Cancer Research.* 1998;4(4):935-939.

11. Bendell JC, Ervin TJ, Senzer NN, et al. Results of the X-PECT study: A phase III randomized double-blind, placebo-controlled study of perifosine plus capecitabine (P-CAP) versus placebo plus capecitabine (CAP) in patients (pts) with refractory metastatic colorectal cancer (mCRC). *Journal of Clinical Oncology.* 2012;30(18_suppl):LBA3501-LBA3501.

12. Blanke CD, Shultz J, Cox J, et al. A double-blind placebo-controlled randomized phase III trial of 5-fluorouracil and leucovorin, plus or minus trimetrexate, in previously untreated patients with advanced colorectal cancer. *Annals of oncology : official journal of the European Society for Medical Oncology.* 2002;13(1):87-91.

13. Blijham G, Wagener T, Wils J, et al. Modulation of high-dose infusional fluorouracil by low-dose methotrexate in patients with advanced or metastatic colorectal cancer: final results of a randomized European Organization for Research and Treatment of Cancer Study. *Journal of clinical oncology : official journal of the American Society of Clinical Oncology.* 1996;14(8):2266-2273.

14. Bobbio-Pallavicini E, Porta C, Moroni M, Spaghi A, Casagranda I, Nastasi G. Folinic acid does improve 5-fluorouracil activity in vivo. Results of a phase III study comparing 5-fluorouracil to 5-fluorouracil and folinic acid in advanced colon cancer patients. *Journal of chemotherapy (Florence, Italy).* 1993;5(1):52-55.

15. Borner MM, Castiglione M, Bacchi M, et al. The impact of adding low-dose leucovorin to monthly 5-fluorouracil in advanced colorectal carcinoma: results of a phase III trial. Swiss Group for Clinical Cancer Research (SAKK). *Annals of oncology : official journal of the European Society for Medical Oncology.* 1998;9(5):535-541.

16. Cardone C, Martinelli E, Troiani T, et al. Exploratory findings from a prematurely closed international, multicentre, academic trial: RAVELLO, a phase III study of regorafenib versus placebo as maintenance therapy after first-line treatment in RAS wild-type metastatic colorectal cancer. *ESMO open.* 2019;4(4):e000519.

17. Carmichael J, Popiela T, Radstone D, et al. Randomized comparative study of tegafur/uracil and oral leucovorin versus parenteral fluorouracil and leucovorin in patients with previously untreated metastatic colorectal cancer. *Journal of clinical oncology : official journal of the American Society of Clinical Oncology.* 2002;20(17):3617-3627.

© 2022 Shen C et al. *JAMA Network Open.*

18. Carrato A, Swieboda-Sadlej A, Staszewska-Skurczynska M, et al. Fluorouracil, leucovorin, and irinotecan plus either sunitinib or placebo in metastatic colorectal cancer: a randomized, phase III trial. *Journal of clinical oncology : official journal of the American Society of Clinical Oncology*. 2013;31(10):1341-1347.

19. Cascinu S, Rosati G, Nasti G, et al. Treatment sequence with either irinotecan/cetuximab followed by FOLFOX-4 or the reverse strategy in metastatic colorectal cancer patients progressing after first-line FOLFIRI/bevacizumab: An Italian Group for the Study of Gastrointestinal Cancer phase III, randomised trial comparing two sequences of therapy in colorectal metastatic patients. *European journal of cancer (Oxford, England : 1990)*. 2017;83:106-115.

20. Cassidy J, Clarke S, Díaz-Rubio E, et al. Randomized phase III study of capecitabine plus oxaliplatin compared with fluorouracil/folinic acid plus oxaliplatin as first-line therapy for metastatic colorectal cancer. *Journal of clinical oncology : official journal of the American Society of Clinical Oncology*. 2008;26(12):2006-2012.

21. Cassidy J, Clarke S, Díaz-Rubio E, et al. XELOX vs FOLFOX-4 as first-line therapy for metastatic colorectal cancer: NO16966 updated results. *British journal of cancer*. 2011;105(1):58-64.

22. Chibaudel B, Bonnetain F, Tournigand C, et al. STRATEGIC-1: A multiple-lines, randomized, open-label GERCOR phase III study in patients with unresectable wild-type RAS metastatic colorectal cancer. *BMC cancer*. 2015;15:496.

23. Chong G, Bhatnagar A, Cunningham D, et al. Phase III trial of 5-fluorouracil and leucovorin plus either 3H1 anti-idiotype monoclonal antibody or placebo in patients with advanced colorectal cancer. *Annals of oncology : official journal of the European Society for Medical Oncology*. 2006;17(3):437-442.

24. Colucci G, Gebbia V, Paoletti G, et al. Phase III randomized trial of FOLFIRI versus FOLFOX4 in the treatment of advanced colorectal cancer: a multicenter study of the Gruppo Oncologico Dell'Italia Meridionale. *Journal of clinical oncology : official journal of the American Society of Clinical Oncology*. 2005;23(22):4866-4875.

25. Comella P, Crucitta E, De Vita F, et al. Addition of either irinotecan or methotrexate to bolus 5-fluorouracil and high-dose folinic acid every 2 weeks in advanced colorectal carcinoma: a randomised study by the Southern Italy Cooperative Oncology Group. *Annals of oncology : official journal of the European Society for Medical Oncology*. 2002;13(6):866-873.

26. Comella P, De Vita F, Mancarella S, et al. Biweekly irinotecan or raltitrexed plus 6S-leucovorin and bolus 5-fluorouracil in advanced colorectal carcinoma: a Southern Italy Cooperative Oncology Group phase II-III randomized trial. *Annals of oncology : official journal of the European Society for Medical Oncology*. 2000;11(10):1323-1333.

© 2022 Shen C et al. *JAMA Network Open.*

27. Comella P, Massidda B, Filippelli G, et al. Oxaliplatin plus high-dose folinic acid and 5-fluorouracil i.v. bolus (OXAFAFU) versus irinotecan plus high-dose folinic acid and 5-fluorouracil i.v. bolus (IRIFAFU) in patients with metastatic colorectal carcinoma: a Southern Italy Cooperative Oncology Group phase III trial. *Annals of oncology : official journal of the European Society for Medical Oncology.* 2005;16(6):878-886.

28. Cremolini C, Loupakis F, Antoniotti C, et al. FOLFOXIRI plus bevacizumab versus FOLFIRI plus bevacizumab as first-line treatment of patients with metastatic colorectal cancer: updated overall survival and molecular subgroup analyses of the open-label, phase 3 TRIBE study. *The Lancet Oncology.* 2015;16(13):1306-1315.

29. Cunningham D, Glimelius B. A phase III study of irinotecan (CPT-11) versus best supportive care in patients with metastatic colorectal cancer who have failed 5-fluorouracil therapy. V301 Study Group. *Seminars in oncology.* 1999;26(1 Suppl 5):6-12.

30. Cunningham D, Lang I, Marcuello E, et al. Bevacizumab plus capecitabine versus capecitabine alone in elderly patients with previously untreated metastatic colorectal cancer (AVEX): an open-label, randomised phase 3 trial. *The Lancet Oncology.* 2013;14(11):1077-1085.

31. Cunningham D, Sirohi B, Pluzanska A, et al. Two different first-line 5-fluorouracil regimens with or without oxaliplatin in patients with metastatic colorectal cancer. *Annals of oncology : official journal of the European Society for Medical Oncology.* 2009;20(2):244-250.

32. Cunningham D, Zalcberg JR, Rath U, et al. 'Tomudex' (ZD1694): results of a randomised trial in advanced colorectal cancer demonstrate efficacy and reduced mucositis and leucopenia. The 'Tomudex' Colorectal Cancer Study Group. *European journal of cancer (Oxford, England : 1990).* 1995;31a(12):1945-1954.

33. de Gramont A, Figer A, Seymour M, et al. Leucovorin and fluorouracil with or without oxaliplatin as first-line treatment in advanced colorectal cancer. *Journal of clinical oncology : official journal of the American Society of Clinical Oncology.* 2000;18(16):2938-2947.

34. Diaz LA, Le DT, Yoshino T, et al. KEYNOTE-177: First-line, open-label, randomized, phase 3 study of pembrolizumab versus investigator-choice chemotherapy for mismatch repair-deficient or microsatellite instability-high metastatic colorectal carcinoma. *Journal of Clinical Oncology.* 2016;34(15_suppl):TPS3639-TPS3639.

35. Díaz-Rubio E, Gómez-España A, Massutí B, et al. First-line XELOX plus bevacizumab followed by XELOX plus bevacizumab or single-agent bevacizumab as maintenance therapy in patients with metastatic colorectal cancer: the phase III MACRO TTD study. *The oncologist.* 2012;17(1):15-25.

36. Díaz-Rubio E, Tabernero J, Gómez-España A, et al. Phase III study of capecitabine plus oxaliplatin compared with continuous-infusion fluorouracil plus oxaliplatin as first-line therapy in metastatic colorectal cancer: final report of the Spanish Cooperative Group for the Treatment of Digestive Tumors Trial. *Journal of clinical oncology : official journal of the American Society of Clinical Oncology*. 2007;25(27):4224-4230.

37. Douillard JY, Cunningham D, Roth AD, et al. Irinotecan combined with fluorouracil compared with fluorouracil alone as first-line treatment for metastatic colorectal cancer: a multicentre randomised trial. *Lancet (London, England)*. 2000;355(9209):1041-1047.

38. Douillard JY, Hoff PM, Skillings JR, et al. Multicenter phase III study of uracil/tegafur and oral leucovorin versus fluorouracil and leucovorin in patients with previously untreated metastatic colorectal cancer. *Journal of clinical oncology : official journal of the American Society of Clinical Oncology*. 2002;20(17):3605-3616.

39. Douillard JY, Siena S, Cassidy J, et al. Final results from PRIME: randomized phase III study of panitumumab with FOLFOX4 for first-line treatment of metastatic colorectal cancer. *Annals of oncology : official journal of the European Society for Medical Oncology*. 2014;25(7):1346-1355.

40. Ducreux M, Bennouna J, Hebbar M, et al. Capecitabine plus oxaliplatin (XELOX) versus 5-fluorouracil/leucovorin plus oxaliplatin (FOLFOX-6) as first-line treatment for metastatic colorectal cancer. *International journal of cancer*. 2011;128(3):682-690.

41. Ducreux M, Malka D, Mendiboure J, et al. Sequential versus combination chemotherapy for the treatment of advanced colorectal cancer (FFCD 2000-05): an open-label, randomised, phase 3 trial. *The Lancet Oncology*. 2011;12(11):1032-1044.

42. Falcone A, Ricci S, Brunetti I, et al. Phase III trial of infusional fluorouracil, leucovorin, oxaliplatin, and irinotecan (FOLFOXIRI) compared with infusional fluorouracil, leucovorin, and irinotecan (FOLFIRI) as first-line treatment for metastatic colorectal cancer: the Gruppo Oncologico Nord Ovest. *Journal of clinical oncology : official journal of the American Society of Clinical Oncology*. 2007;25(13):1670-1676.

43. Fischer von Weikersthal L, Schalhorn A, Stauch M, et al. Phase III trial of irinotecan plus infusional 5-fluorouracil/folinic acid versus irinotecan plus oxaliplatin as first-line treatment of advanced colorectal cancer. *European journal of cancer (Oxford, England : 1990)*. 2011;47(2):206-214.

44. Fisher GA. A phase III study of xilonix in refractory colorectal cancer patients with weight loss. *Journal of Clinical Oncology*. 2015;33(3_suppl):685-685.

© 2022 Shen C et al. *JAMA Network Open.*

45. Fuchs CS, Marshall J, Barrueco J. Randomized, controlled trial of irinotecan plus infusional, bolus, or oral fluoropyrimidines in first-line treatment of metastatic colorectal cancer: updated results from the BICC-C study. *Journal of clinical oncology : official journal of the American Society of Clinical Oncology*. 2008;26(4):689-690.

46. Fuchs CS, Marshall J, Mitchell E, et al. Randomized, controlled trial of irinotecan plus infusional, bolus, or oral fluoropyrimidines in first-line treatment of metastatic colorectal cancer: results from the BICC-C Study. *Journal of clinical oncology : official journal of the American Society of Clinical Oncology*. 2007;25(30):4779-4786.

47. Fuchs CS, Moore MR, Harker G, Villa L, Rinaldi D, Hecht JR. Phase III comparison of two irinotecan dosing regimens in second-line therapy of metastatic colorectal cancer. *Journal of clinical oncology : official journal of the American Society of Clinical Oncology*. 2003;21(5):807-814.

48. Gamelin E, Delva R, Jacob J, et al. Individual fluorouracil dose adjustment based on pharmacokinetic follow-up compared with conventional dosage: results of a multicenter randomized trial of patients with metastatic colorectal cancer. *Journal of clinical oncology : official journal of the American Society of Clinical Oncology*. 2008;26(13):2099-2105.

49. Giacchetti S, Bjarnason G, Garufi C, et al. Phase III trial comparing 4-day chronomodulated therapy versus 2-day conventional delivery of fluorouracil, leucovorin, and oxaliplatin as first-line chemotherapy of metastatic colorectal cancer: the European Organisation for Research and Treatment of Cancer Chronotherapy Group. *Journal of clinical oncology : official journal of the American Society of Clinical Oncology*. 2006;24(22):3562-3569.

50. Giacchetti S, Perpoint B, Zidani R, et al. Phase III multicenter randomized trial of oxaliplatin added to chronomodulated fluorouracil-leucovorin as first-line treatment of metastatic colorectal cancer. *Journal of clinical oncology : official journal of the American Society of Clinical Oncology*. 2000;18(1):136-147.

51. Giantonio BJ, Catalano PJ, Meropol NJ, et al. Bevacizumab in combination with oxaliplatin, fluorouracil, and leucovorin (FOLFOX4) for previously treated metastatic colorectal cancer: results from the Eastern Cooperative Oncology Group Study E3200. *Journal of clinical oncology : official journal of the American Society of Clinical Oncology*. 2007;25(12):1539-1544.

52. Glimelius B, Sørbye H, Balteskard L, et al. A randomized phase III multicenter trial comparing irinotecan in combination with the Nordic bolus 5-FU and folinic acid schedule or the bolus/infused de Gramont schedule (Lv5FU2) in patients with metastatic colorectal cancer. *Annals of oncology : official journal of the European Society for Medical Oncology*. 2008;19(5):909-914.

53. Goel S, Wyrwicz L, Choi M, et al. Phase III double-blinded, placebo-controlled study of MABp1 for improving survival in metastatic colorectal cancer. *Journal of Clinical Oncology*. 2016;34(4_suppl):TPS784-TPS784.

© 2022 Shen C et al. *JAMA Network Open.*

54. Goldberg RM, Hatfield AK, Kahn M, et al. Prospectively randomized North Central Cancer Treatment Group trial of intensive-course fluorouracil combined with the l-isomer of intravenous leucovorin, oral leucovorin, or intravenous leucovorin for the treatment of advanced colorectal cancer. *Journal of clinical oncology : official journal of the American Society of Clinical Oncology*. 1997;15(11):3320-3329.

55. Goldberg RM, Moertel CG, Wieand HS, et al. A phase III evaluation of a somatostatin analogue (octreotide) in the treatment of patients with asymptomatic advanced colon carcinoma. North Central Cancer Treatment Group and the Mayo Clinic. *Cancer*. 1995;76(6):961-966.

56. Goldberg RM, Sargent DJ, Morton RF, et al. A randomized controlled trial of fluorouracil plus leucovorin, irinotecan, and oxaliplatin combinations in patients with previously untreated metastatic colorectal cancer. *Journal of clinical oncology : official journal of the American Society of Clinical Oncology*. 2004;22(1):23-30.

57. Greco FA, Figlin R, York M, et al. Phase III randomized study to compare interferon alfa-2a in combination with fluorouracil versus fluorouracil alone in patients with advanced colorectal cancer. *Journal of clinical oncology : official journal of the American Society of Clinical Oncology*. 1996;14(10):2674-2681.

58. Grothey A, Shah MA, Yoshino T, et al. CanStem303C trial: A phase III study of napabucasin (BBI-608) in combination with 5-fluorouracil (5-FU), leucovorin, irinotecan (FOLFIRI) in adult patients with previously treated metastatic colorectal cancer (mCRC). *Journal of Clinical Oncology*. 2017;35(15_suppl):TPS3619-TPS3619.

59. Grothey A, Van Cutsem E, Sobrero A, et al. Regorafenib monotherapy for previously treated metastatic colorectal cancer (CORRECT): an international, multicentre, randomised, placebo-controlled, phase 3 trial. *Lancet (London, England)*. 2013;381(9863):303-312.

60. Guan ZZ, Xu JM, Luo RC, et al. Efficacy and safety of bevacizumab plus chemotherapy in Chinese patients with metastatic colorectal cancer: a randomized phase III ARTIST trial. *Chinese journal of cancer*. 2011;30(10):682-689.

61. Hagman H, Frödin JE, Berglund Å, et al. A randomized study of KRAS-guided maintenance therapy with bevacizumab, erlotinib or metronomic capecitabine after first-line induction treatment of metastatic colorectal cancer: the Nordic ACT2 trial. *Annals of oncology : official journal of the European Society for Medical Oncology*. 2016;27(1):140-147.

62. Haller DG, Rothenberg ML, Wong AO, et al. Oxaliplatin plus irinotecan compared with irinotecan alone as second-line treatment after single-agent fluoropyrimidine therapy for metastatic colorectal carcinoma. *Journal of clinical oncology : official journal of the American Society of Clinical Oncology*. 2008;26(28):4544-4550.

© 2022 Shen C et al. *JAMA Network Open.*

63. Hansen RM, Ryan L, Anderson T, et al. Phase III study of bolus versus infusion fluorouracil with or without cisplatin in advanced colorectal cancer. *Journal of the National Cancer Institute*. 1996;88(10):668-674.

64. Hausmaninger H, Moser R, Samonigg H, et al. Biochemical modulation of 5-fluorouracil by leucovorin with or without interferon-alpha-2c in patients with advanced colorectal cancer: final results of a randomised phase III study. *European journal of cancer (Oxford, England : 1990)*. 1999;35(3):380-385.

65. Hecht JR, Mitchell E, Chidiac T, et al. A randomized phase IIIB trial of chemotherapy, bevacizumab, and panitumumab compared with chemotherapy and bevacizumab alone for metastatic colorectal cancer. *Journal of clinical oncology : official journal of the American Society of Clinical Oncology*. 2009;27(5):672-680.

66. Hecht JR, Trarbach T, Hainsworth JD, et al. Randomized, placebo-controlled, phase III study of first-line oxaliplatin-based chemotherapy plus PTK787/ZK 222584, an oral vascular endothelial growth factor receptor inhibitor, in patients with metastatic colorectal adenocarcinoma. *Journal of clinical oncology : official journal of the American Society of Clinical Oncology*. 2011;29(15):1997-2003.

67. Hegewisch-Becker S, Graeven U, Lerchenmüller CA, et al. Maintenance strategies after first-line oxaliplatin plus fluoropyrimidine plus bevacizumab for patients with metastatic colorectal cancer (AIO 0207): a randomised, non-inferiority, open-label, phase 3 trial. *The Lancet Oncology*. 2015;16(13):1355-1369.

68. Heinemann V, von Weikersthal LF, Decker T, et al. FOLFIRI plus cetuximab versus FOLFIRI plus bevacizumab as first-line treatment for patients with metastatic colorectal cancer (FIRE-3): a randomised, open-label, phase 3 trial. *The Lancet Oncology*. 2014;15(10):1065-1075.

69. Heys SD, Eremin O, Ruggeri EM, et al. A phase III study of recombinant interleukin-2, 5-fluorouracil and leucovorin versus 5-fluorouracil and leucovorin in patients with unresectable or metastatic colorectal carcinoma. *European journal of cancer (Oxford, England : 1990)*. 1995;31a(1):19-25.

70. Hickish T, Andre T, Wyrwicz L, et al. MABp1 as a novel antibody treatment for advanced colorectal cancer: a randomised, double-blind, placebo-controlled, phase 3 study. *The Lancet Oncology*. 2017;18(2):192-201.

71. Hill M, Norman A, Cunningham D, et al. Royal Marsden phase III trial of fluorouracil with or without interferon alfa-2b in advanced colorectal cancer. *Journal of clinical oncology : official journal of the American Society of Clinical Oncology*. 1995;13(6):1297-1302.

© 2022 Shen C et al. *JAMA Network Open.*

72. Hochster HS, Grothey A, Hart L, et al. Improved time to treatment failure with an intermittent oxaliplatin strategy: results of CONcePT. *Annals of oncology : official journal of the European Society for Medical Oncology.* 2014;25(6):1172-1178.

73. Hoff PM, Ansari R, Batist G, et al. Comparison of oral capecitabine versus intravenous fluorouracil plus leucovorin as first-line treatment in 605 patients with metastatic colorectal cancer: results of a randomized phase III study. *Journal of clinical oncology : official journal of the American Society of Clinical Oncology.* 2001;19(8):2282-2292.

74. Hoff PM, Hochhaus A, Pestalozzi BC, et al. Cediranib plus FOLFOX/CAPOX versus placebo plus FOLFOX/CAPOX in patients with previously untreated metastatic colorectal cancer: a randomized, double-blind, phase III study (HORIZON II). *Journal of clinical oncology : official journal of the American Society of Clinical Oncology.* 2012;30(29):3596-3603.

75. Hong YS, Park YS, Lim HY, et al. S-1 plus oxaliplatin versus capecitabine plus oxaliplatin for first-line treatment of patients with metastatic colorectal cancer: a randomised, non-inferiority phase 3 trial. *The Lancet Oncology.* 2012;13(11):1125-1132.

76. Hospers GA, Schaapveld M, Nortier JW, et al. Randomised Phase III study of biweekly 24-h infusion of high-dose 5FU with folinic acid and oxaliplatin versus monthly plus 5-FU/folinic acid in first-line treatment of advanced colorectal cancer. *Annals of oncology : official journal of the European Society for Medical Oncology.* 2006;17(3):443-449.

77. Huhn R, Lowe J, Grady M, Taitt C, Braun A. P-312 A Phase 3 Open-Label, Randomized, Multicenter Study of Imprime PGG in Combination with Cetuximab in Patients with KRAS Wild Type Metastatic Colorectal Cancer. *Annals of Oncology.* 2015;26:iv92.

78. Huiskens J, van Gulik TM, van Lienden KP, et al. Treatment strategies in colorectal cancer patients with initially unresectable liver-only metastases, a study protocol of the randomised phase 3 CAIRO5 study of the Dutch Colorectal Cancer Group (DCCG). *BMC cancer.* 2015;15:365.

79. Hurwitz H, Fehrenbacher L, Novotny W, et al. Bevacizumab plus irinotecan, fluorouracil, and leucovorin for metastatic colorectal cancer. *The New England journal of medicine.* 2004;350(23):2335-2342.

80. Hurwitz H, Mitchell EP, Cartwright T, et al. A randomized, phase II trial of standard triweekly compared with dose-dense biweekly capecitabine plus oxaliplatin plus bevacizumab as first-line treatment for metastatic colorectal cancer: XELOX-A-DVS (dense versus standard). *The oncologist.* 2012;17(7):937-946.

81. Iwamoto S, Takahashi T, Tamagawa H, et al. FOLFIRI plus bevacizumab as second-line therapy in patients with metastatic colorectal cancer after first-line bevacizumab plus oxaliplatin-based therapy: the randomized phase III EAGLE study. *Annals of oncology : official journal of the European Society for Medical Oncology.* 2015;26(7):1427-1433.

© 2022 Shen C et al. *JAMA Network Open.*

82. Johnsson A, Hagman H, Frödin JE, et al. A randomized phase III trial on maintenance treatment with bevacizumab alone or in combination with erlotinib after chemotherapy and bevacizumab in metastatic colorectal cancer: the Nordic ACT Trial. *Annals of oncology : official journal of the European Society for Medical Oncology*. 2013;24(9):2335-2341.

83. Jones DV, Jr., Winn RJ, Brown BW, et al. Randomized phase III study of 5-fluorouracil plus high dose folinic acid versus 5-fluorouracil plus folinic acid plus methyl-lomustine for patients with advanced colorectal cancer. *Cancer*. 1995;76(10):1709-1714.

84. Jonker DJ, Nott L, Yoshino T, et al. Napabucasin versus placebo in refractory advanced colorectal cancer: a randomised phase 3 trial. *The lancet Gastroenterology & hepatology*. 2018;3(4):263-270.

85. Kalofonos HP, Nicolaides C, Samantas E, et al. A phase III study of 5-fluorouracil versus 5-fluorouracil plus interferon alpha 2b versus 5-fluorouracil plus leucovorin in patients with advanced colorectal cancer: a Hellenic Cooperative Oncology Group (HeCOG) study. *American journal of clinical oncology*. 2002;25(1):23-30.

86. Kim GP, Sargent DJ, Mahoney MR, et al. Phase III noninferiority trial comparing irinotecan with oxaliplatin, fluorouracil, and leucovorin in patients with advanced colorectal carcinoma previously treated with fluorouracil: N9841. *Journal of clinical oncology : official journal of the American Society of Clinical Oncology*. 2009;27(17):2848-2854.

87. Kim ST, Hong YS, Lim HY, et al. S-1 plus oxaliplatin versus capecitabine plus oxaliplatin for the first-line treatment of patients with metastatic colorectal cancer: updated results from a phase 3 trial. *BMC cancer*. 2014;14:883.

88. Kim TW, Elme A, Kusic Z, et al. A phase 3 trial evaluating panitumumab plus best supportive care vs best supportive care in chemorefractory wild-type KRAS or RAS metastatic colorectal cancer. *British journal of cancer*. 2016;115(10):1206-1214.

89. Koeberle D, Betticher DC, von Moos R, et al. Bevacizumab continuation versus no continuation after first-line chemotherapy plus bevacizumab in patients with metastatic colorectal cancer: a randomized phase III non-inferiority trial (SAKK 41/06). *Annals of oncology : official journal of the European Society for Medical Oncology*. 2015;26(4):709-714.

90. Köhne CH, De Greve J, Hartmann JT, et al. Irinotecan combined with infusional 5-fluorouracil/folinic acid or capecitabine plus celecoxib or placebo in the first-line treatment of patients with metastatic colorectal cancer. EORTC study 40015. *Annals of oncology : official journal of the European Society for Medical Oncology*. 2008;19(5):920-926.

91. Köhne CH, van Cutsem E, Wils J, et al. Phase III study of weekly high-dose infusional fluorouracil plus folinic acid with or without irinotecan in patients with metastatic colorectal cancer: European Organisation for Research and Treatment of Cancer

© 2022 Shen C et al. *JAMA Network Open.*

Gastrointestinal Group Study 40986. *Journal of clinical oncology : official journal of the American Society of Clinical Oncology*. 2005;23(22):4856-4865.

92. Köhne CH, Wils J, Lorenz M, et al. Randomized phase III study of high-dose fluorouracil given as a weekly 24-hour infusion with or without leucovorin versus bolus fluorouracil plus leucovorin in advanced colorectal cancer: European organization of Research and Treatment of Cancer Gastrointestinal Group Study 40952. *Journal of clinical oncology : official journal of the American Society of Clinical Oncology*. 2003;21(20):3721-3728.

93. Komatsu Y, Ishioka C, Shimada K, et al. Study protocol of the TRICOLORE trial: a randomized phase III study of oxaliplatin-based chemotherapy versus combination chemotherapy with S-1, irinotecan, and bevacizumab as first-line therapy for metastatic colorectal cancer. *BMC cancer*. 2015;15:626.

94. Koopman M, Antonini NF, Douma J, et al. Sequential versus combination chemotherapy with capecitabine, irinotecan, and oxaliplatin in advanced colorectal cancer (CAIRO): a phase III randomised controlled trial. *Lancet (London, England)*. 2007;370(9582):135-142.

95. Kosmidis PA, Tsavaris N, Skarlos D, et al. Fluorouracil and leucovorin with or without interferon alfa-2b in advanced colorectal cancer: analysis of a prospective randomized phase III trial. Hellenic Cooperative Oncology Group. *Journal of clinical oncology : official journal of the American Society of Clinical Oncology*. 1996;14(10):2682-2687.

96. Kwakman JJM, Simkens LHJ, van Rooijen JM, et al. Randomized phase III trial of S-1 versus capecitabine in the first-line treatment of metastatic colorectal cancer: SALTO study by the Dutch Colorectal Cancer Group. *Annals of oncology : official journal of the European Society for Medical Oncology*. 2017;28(6):1288-1293.

97. Labianca R, Cascinu S, Frontini L, et al. High-versus low-dose levo-leucovorin as a modulator of 5-fluorouracil in advanced colorectal cancer: a 'GISCAD' phase III study. Italian Group for the Study of Digestive Tract Cancer. *Annals of oncology : official journal of the European Society for Medical Oncology*. 1997;8(2):169-174.

98. Lal R, Dickson J, Cunningham D, et al. A randomized trial comparing defined-duration with continuous irinotecan until disease progression in fluoropyrimidine and thymidylate synthase inhibitor-resistant advanced colorectal cancer. *Journal of clinical oncology : official journal of the American Society of Clinical Oncology*. 2004;22(15):3023-3031.

99. Li J, Qin S, Xu R, et al. Regorafenib plus best supportive care versus placebo plus best supportive care in Asian patients with previously treated metastatic colorectal cancer (CONCUR): a randomised, double-blind, placebo-controlled, phase 3 trial. *The Lancet Oncology*. 2015;16(6):619-629.

© 2022 Shen C et al. *JAMA Network Open.*

100. Lim SH, Kim TW, Hong YS, et al. A randomised, double-blind, placebo-controlled multi-centre phase III trial of XELIRI/FOLFIRI plus simvastatin for patients with metastatic colorectal cancer. *British journal of cancer.* 2015;113(10):1421-1426.

101. Luo HY, Li YH, Wang W, et al. Single-agent capecitabine as maintenance therapy after induction of XELOX (or FOLFOX) in first-line treatment of metastatic colorectal cancer: randomized clinical trial of efficacy and safety. *Annals of oncology : official journal of the European Society for Medical Oncology.* 2016;27(6):1074-1081.

102. Masi G, Salvatore L, Boni L, et al. Continuation or reintroduction of bevacizumab beyond progression to first-line therapy in metastatic colorectal cancer: final results of the randomized BEBYP trial. *Annals of oncology : official journal of the European Society for Medical Oncology.* 2015;26(4):724-730.

103. Maughan TS, Adams RA, Smith CG, et al. Addition of cetuximab to oxaliplatin-based first-line combination chemotherapy for treatment of advanced colorectal cancer: results of the randomised phase 3 MRC COIN trial. *Lancet (London, England).* 2011;377(9783):2103-2114.

104. Mayer RJ, Van Cutsem E, Falcone A, et al. Randomized trial of TAS-102 for refractory metastatic colorectal cancer. *The New England journal of medicine.* 2015;372(20):1909-1919.

105. Middleton G, Brown S, Lowe C, et al. A randomised phase III trial of the pharmacokinetic biomodulation of irinotecan using oral ciclosporin in advanced colorectal cancer: results of the Panitumumab, Irinotecan & Ciclosporin in COLOrectal cancer therapy trial (PICCOLO). *European journal of cancer (Oxford, England : 1990).* 2013;49(16):3507-3516.

106. Muro K, Boku N, Shimada Y, et al. Irinotecan plus S-1 (IRIS) versus fluorouracil and folinic acid plus irinotecan (FOLFIRI) as second-line chemotherapy for metastatic colorectal cancer: a randomised phase 2/3 non-inferiority study (FIRIS study). *The Lancet Oncology.* 2010;11(9):853-860.

107. Muro K, Kim TW, Park YS, et al. A multinational, randomized, phase III trial of XELIRI (+bevacizumab) versus FOLFIRI (+bevacizumab) as the second-line chemotherapy for metastatic colorectal cancer: Asian XELIRI project (AXEPT). *Journal of Clinical Oncology.* 2016;34(4_suppl):TPS780-TPS780.

108. Muro K, Uetake H, Tsuchihara K, et al. PARADIGM study: A multicenter, randomized, phase III study of mFOLFOX6 plus panitumumab or bevacizumab as first-line treatment in patients with RAS (KRAS/NRAS) wild-type metastatic colorectal cancer. *Journal of Clinical Oncology.* 2016;34(15_suppl):TPS3625-TPS3625.

© 2022 Shen C et al. *JAMA Network Open.*

109. Nct. Phase III Randomized Study of 5-FU, CoFactor, and Avastin vs. 5-FU, LV and Avastin for First-Line Colorectal Cancer. [*https://clinicaltrialsgov/show/NCT00337389*](https://clinicaltrialsgov/show/NCT00337389). 2006.

110. Opel DJ. Physician, patient, parent: where exactly is the line? *The Hastings Center report.* 2012;42(6):14-18.

111. Passardi A, Nanni O, Tassinari D, et al. Effectiveness of bevacizumab added to standard chemotherapy in metastatic colorectal cancer: final results for first-line treatment from the ITACa randomized clinical trial. *Annals of oncology : official journal of the European Society for Medical Oncology.* 2015;26(6):1201-1207.

112. Passardi A, Scarpi E, Gelsomino F, et al. Impact of second-line cetuximab-containing therapy in patients with KRAS wild-type metastatic colorectal cancer: results from the ITACa randomized clinical trial. *Scientific reports.* 2017;7(1):10426.

113. Pectasides D, Papaxoinis G, Kalogeras KT, et al. XELIRI-bevacizumab versus FOLFIRI-bevacizumab as first-line treatment in patients with metastatic colorectal cancer: a Hellenic Cooperative Oncology Group phase III trial with collateral biomarker analysis. *BMC cancer.* 2012;12:271.

114. Peeters M, Price TJ, Cervantes A, et al. Final results from a randomized phase 3 study of FOLFIRI {+/-} panitumumab for second-line treatment of metastatic colorectal cancer. *Annals of oncology : official journal of the European Society for Medical Oncology.* 2014;25(1):107-116.

115. Peeters M, Price TJ, Cervantes A, et al. Randomized phase III study of panitumumab with fluorouracil, leucovorin, and irinotecan (FOLFIRI) compared with FOLFIRI alone as second-line treatment in patients with metastatic colorectal cancer. *Journal of clinical oncology : official journal of the American Society of Clinical Oncology.* 2010;28(31):4706-4713.

116. Petrelli N, Douglass HO, Jr., Herrera L, et al. The modulation of fluorouracil with leucovorin in metastatic colorectal carcinoma: a prospective randomized phase III trial. Gastrointestinal Tumor Study Group. *Journal of clinical oncology : official journal of the American Society of Clinical Oncology.* 1989;7(10):1419-1426.

117. Pinter T, Klippel Z, Cesas A, et al. A Phase III, Randomized, Double-Blind, Placebo-Controlled Trial of Pegfilgrastim in Patients Receiving First-Line FOLFOX/Bevacizumab or FOLFIRI/Bevacizumab for Locally Advanced or Metastatic Colorectal Cancer: Final Results of the Pegfilgrastim and Anti-VEGF Evaluation Study (PAVES). *Clinical colorectal cancer.* 2017;16(2):103-114.e103.

© 2022 Shen C et al. *JAMA Network Open.*

118. Porschen R, Arkenau HT, Kubicka S, et al. Phase III study of capecitabine plus oxaliplatin compared with fluorouracil and leucovorin plus oxaliplatin in metastatic colorectal cancer: a final report of the AIO Colorectal Study Group. *Journal of clinical oncology : official journal of the American Society of Clinical Oncology.* 2007;25(27):4217-4223.

119. Price T, Kim TW, Li J, et al. Final results and outcomes by prior bevacizumab exposure, skin toxicity, and hypomagnesaemia from ASPECCT: randomized phase 3 non-inferiority study of panitumumab versus cetuximab in chemorefractory wild-type KRAS exon 2 metastatic colorectal cancer. *European journal of cancer (Oxford, England : 1990).* 2016;68:51-59.

120. Price TJ, Peeters M, Kim TW, et al. Panitumumab versus cetuximab in patients with chemotherapy-refractory wild-type KRAS exon 2 metastatic colorectal cancer (ASPECCT): a randomised, multicentre, open-label, non-inferiority phase 3 study. *The Lancet Oncology.* 2014;15(6):569-579.

121. Price TJ, Ross PJ, Hickish T, et al. Phase III study of mitomycin-C with protracted venous infusion or circadian-timed infusion of 5-fluorouracil in advanced colorectal carcinoma. *Clinical colorectal cancer.* 2004;3(4):235-242.

122. Qin S, Guo W, Xu J-M, et al. Final overall survival (OS) analysis of first-line (1L) FOLFOX-4 ± cetuximab (cet) in patients (pts) with RAS wild-type (wt) metastatic colorectal cancer (mCRC) in the phase 3 TAILOR trial. *Journal of Clinical Oncology.* 2018;36(15_suppl):3521-3521.

123. Rao S, Cunningham D, de Gramont A, et al. Phase III double-blind placebo-controlled study of farnesyl transferase inhibitor R115777 in patients with refractory advanced colorectal cancer. *Journal of clinical oncology : official journal of the American Society of Clinical Oncology.* 2004;22(19):3950-3957.

124. Rothenberg ML, Cox JV, Butts C, et al. Capecitabine plus oxaliplatin (XELOX) versus 5-fluorouracil/folinic acid plus oxaliplatin (FOLFOX-4) as second-line therapy in metastatic colorectal cancer: a randomized phase III noninferiority study. *Annals of oncology : official journal of the European Society for Medical Oncology.* 2008;19(10):1720-1726.

125. Rothenberg ML, Oza AM, Bigelow RH, et al. Superiority of oxaliplatin and fluorouracil-leucovorin compared with either therapy alone in patients with progressive colorectal cancer after irinotecan and fluorouracil-leucovorin: interim results of a phase III trial. *Journal of clinical oncology : official journal of the American Society of Clinical Oncology.* 2003;21(11):2059-2069.

126. Rougier P, Paillot B, LaPlanche A, et al. 5-Fluorouracil (5-FU) continuous intravenous infusion compared with bolus administration. Final results of a randomised trial in metastatic colorectal cancer. *European journal of cancer (Oxford, England : 1990).* 1997;33(11):1789-1793.

© 2022 Shen C et al. *JAMA Network Open.*

127. Rougier P, Van Cutsem E, Bajetta E, et al. Randomised trial of irinotecan versus fluorouracil by continuous infusion after fluorouracil failure in patients with metastatic colorectal cancer. *Lancet (London, England).* 1998;352(9138):1407-1412.

128. Saltz L, Badarinath S, Dakhil S, et al. Phase III trial of cetuximab, bevacizumab, and 5-fluorouracil/leucovorin vs. FOLFOX-bevacizumab in colorectal cancer. *Clinical colorectal cancer.* 2012;11(2):101-111.

129. Saltz LB, Clarke S, Díaz-Rubio E, et al. Bevacizumab in combination with oxaliplatin-based chemotherapy as first-line therapy in metastatic colorectal cancer: a randomized phase III study. *Journal of clinical oncology : official journal of the American Society of Clinical Oncology.* 2008;26(12):2013-2019.

130. Saltz LB, Cox JV, Blanke C, et al. Irinotecan plus fluorouracil and leucovorin for metastatic colorectal cancer. Irinotecan Study Group. *The New England journal of medicine.* 2000;343(13):905-914.

131. Scheithauer W, Kornek G, Marczell A, et al. Fluorouracil plus racemic leucovorin versus fluorouracil combined with the pure l-isomer of leucovorin for the treatment of advanced colorectal cancer: a randomized phase III study. *Journal of clinical oncology : official journal of the American Society of Clinical Oncology.* 1997;15(3):908-914.

132. Schilsky RL, Levin J, West WH, et al. Randomized, open-label, phase III study of a 28-day oral regimen of eniluracil plus fluorouracil versus intravenous fluorouracil plus leucovorin as first-line therapy in patients with metastatic/advanced colorectal cancer. *Journal of clinical oncology : official journal of the American Society of Clinical Oncology.* 2002;20(6):1519-1526.

133. Schmoll HJ, Cunningham D, Sobrero A, et al. Cediranib with mFOLFOX6 versus bevacizumab with mFOLFOX6 as first-line treatment for patients with advanced colorectal cancer: a double-blind, randomized phase III study (HORIZON III). *Journal of clinical oncology : official journal of the American Society of Clinical Oncology.* 2012;30(29):3588-3595.

134. Sclafani F, Kim TY, Cunningham D, et al. A Randomized Phase II/III Study of Dalotuzumab in Combination With Cetuximab and Irinotecan in Chemorefractory, KRAS Wild-Type, Metastatic Colorectal Cancer. *Journal of the National Cancer Institute.* 2015;107(12):djv258.

135. Simkens LH, van Tinteren H, May A, et al. Maintenance treatment with capecitabine and bevacizumab in metastatic colorectal cancer (CAIRO3): a phase 3 randomised controlled trial of the Dutch Colorectal Cancer Group. *Lancet (London, England).* 2015;385(9980):1843-1852.

136. Siu LL, Shapiro JD, Jonker DJ, et al. Phase III randomized, placebo-controlled study of cetuximab plus brivanib alaninate versus cetuximab plus placebo in patients with metastatic, chemotherapy-refractory, wild-type K-RAS colorectal carcinoma: the NCIC

© 2022 Shen C et al. *JAMA Network Open.*

Clinical Trials Group and AGITG CO.20 Trial. *Journal of clinical oncology : official journal of the American Society of Clinical Oncology*. 2013;31(19):2477-2484.

137. Sobrero AF, Maurel J, Fehrenbacher L, et al. EPIC: phase III trial of cetuximab plus irinotecan after fluoropyrimidine and oxaliplatin failure in patients with metastatic colorectal cancer. *Journal of clinical oncology : official journal of the American Society of Clinical Oncology*. 2008;26(14):2311-2319.

138. Souglakos J, Androulakis N, Syrigos K, et al. FOLFOXIRI (folinic acid, 5-fluorouracil, oxaliplatin and irinotecan) vs FOLFIRI (folinic acid, 5-fluorouracil and irinotecan) as first-line treatment in metastatic colorectal cancer (MCC): a multicentre randomised phase III trial from the Hellenic Oncology Research Group (HORG). *British journal of cancer*. 2006;94(6):798-805.

139. Stathopoulos GP, Batziou C, Trafalis D, et al. Treatment of colorectal cancer with and without bevacizumab: a phase III study. *Oncology*. 2010;78(5-6):376-381.

140. Tabernero J, Yoshino T, Cohn AL, et al. Ramucirumab versus placebo in combination with second-line FOLFIRI in patients with metastatic colorectal carcinoma that progressed during or after first-line therapy with bevacizumab, oxaliplatin, and a fluoropyrimidine (RAISE): a randomised, double-blind, multicentre, phase 3 study. *The Lancet Oncology*. 2015;16(5):499-508.

141. Tebbutt NC, Wilson K, Gebski VJ, et al. Capecitabine, bevacizumab, and mitomycin in first-line treatment of metastatic colorectal cancer: results of the Australasian Gastrointestinal Trials Group Randomized Phase III MAX Study. *Journal of clinical oncology : official journal of the American Society of Clinical Oncology*. 2010;28(19):3191-3198.

142. Tol J, Koopman M, Cats A, et al. Chemotherapy, bevacizumab, and cetuximab in metastatic colorectal cancer. *The New England journal of medicine*. 2009;360(6):563-572.

143. Tournigand C, André T, Achille E, et al. FOLFIRI followed by FOLFOX6 or the reverse sequence in advanced colorectal cancer: a randomized GERCOR study. *Journal of clinical oncology : official journal of the American Society of Clinical Oncology*. 2004;22(2):229-237.

144. Tournigand C, Chibaudel B, Samson B, et al. Bevacizumab with or without erlotinib as maintenance therapy in patients with metastatic colorectal cancer (GERCOR DREAM; OPTIMOX3): a randomised, open-label, phase 3 trial. *The Lancet Oncology*. 2015;16(15):1493-1505.

© 2022 Shen C et al. *JAMA Network Open.*

145. Tsavaris N, Kosmas C, Skopelitis H, et al. Sequential administration of 5-fluorouracil (5FU)/leucovorin (LV) followed by irinotecan (CPT-11) at relapse versus CPT-11 followed by 5-FU/LV in advanced colorectal carcinoma. A phase III randomized study. *Chemotherapy.* 2007;53(4):282-291.

146. Tveit KM, Guren T, Glimelius B, et al. Phase III trial of cetuximab with continuous or intermittent fluorouracil, leucovorin, and oxaliplatin (Nordic FLOX) versus FLOX alone in first-line treatment of metastatic colorectal cancer: the NORDIC-VII study. *Journal of clinical oncology : official journal of the American Society of Clinical Oncology.* 2012;30(15):1755-1762.

147. Unger C, Häring B, Kruse A, et al. Double-blind randomised placebo-controlled phase III study of an E. coli extract plus 5-fluorouracil versus 5-fluorouracil in patients with advanced colorectal cancer. *Arzneimittel-Forschung.* 2001;51(4):332-338.

148. Van Cutsem E, Bajetta E, Valle J, et al. Randomized, placebo-controlled, phase III study of oxaliplatin, fluorouracil, and leucovorin with or without PTK787/ZK 222584 in patients with previously treated metastatic colorectal adenocarcinoma. *Journal of clinical oncology : official journal of the American Society of Clinical Oncology.* 2011;29(15):2004-2010.

149. Van Cutsem E, Blijham GH. Irinotecan versus infusional 5-fluorouracil: a phase III study in metastatic colorectal cancer following failure on first-line 5-fluorouracil. V302 Study Group. *Seminars in oncology.* 1999;26(1 Suppl 5):13-20.

150. Van Cutsem E, Martinelli E, Cascinu S, et al. Regorafenib for Patients with Metastatic Colorectal Cancer Who Progressed After Standard Therapy: Results of the Large, Single-Arm, Open-Label Phase IIIb CONSIGN Study. *The oncologist.* 2019;24(2):185-192.

151. Van Cutsem E, Peeters M, Siena S, et al. Open-label phase III trial of panitumumab plus best supportive care compared with best supportive care alone in patients with chemotherapy-refractory metastatic colorectal cancer. *Journal of clinical oncology : official journal of the American Society of Clinical Oncology.* 2007;25(13):1658-1664.

152. Van Cutsem E, Tabernero J, Lakomy R, et al. Addition of aflibercept to fluorouracil, leucovorin, and irinotecan improves survival in a phase III randomized trial in patients with metastatic colorectal cancer previously treated with an oxaliplatin-based regimen. *Journal of clinical oncology : official journal of the American Society of Clinical Oncology.* 2012;30(28):3499-3506.

153. Van Cutsem E, Twelves C, Cassidy J, et al. Oral capecitabine compared with intravenous fluorouracil plus leucovorin in patients with metastatic colorectal cancer: results of a large phase III study. *Journal of clinical oncology : official journal of the American Society of Clinical Oncology.* 2001;19(21):4097-4106.

© 2022 Shen C et al. *JAMA Network Open.*

154. Van Cutsem E, Yoshino T, Lenz HJ, et al. Nintedanib for the treatment of patients with refractory metastatic colorectal cancer (LUME-Colon 1): a phase III, international, randomized, placebo-controlled study. *Annals of oncology : official journal of the European Society for Medical Oncology*. 2018;29(9):1955-1963.

155. Venook AP, Niedzwiecki D, Lenz HJ, et al. Effect of First-Line Chemotherapy Combined With Cetuximab or Bevacizumab on Overall Survival in Patients With KRAS Wild-Type Advanced or Metastatic Colorectal Cancer: A Randomized Clinical Trial. *Jama*. 2017;317(23):2392-2401.

156. Wainberg Z, Hecht JR. A phase III randomized, open-label, controlled trial of chemotherapy and bevacizumab with or without panitumumab in the first-line treatment of patients with metastatic colorectal cancer. *Clinical colorectal cancer*. 2006;5(5):363-367.

157. Wils J, Blijham GH, Wagener T, et al. High-dose 5-fluorouracil plus low dose methotrexate plus or minus low-dose PALA in advanced colorectal cancer: a randomised phase II-III trial of the EORTC Gastrointestinal Group. *European journal of cancer (Oxford, England : 1990)*. 2003;39(3):346-352.

158. Xu J, Kim TW, Shen L, et al. Results of a Randomized, Double-Blind, Placebo-Controlled, Phase III Trial of Trifluridine/Tipiracil (TAS-102) Monotherapy in Asian Patients With Previously Treated Metastatic Colorectal Cancer: The TERRA Study. *Journal of clinical oncology : official journal of the American Society of Clinical Oncology*. 2018;36(4):350-358.

159. Yalcin S, Uslu R, Dane F, et al. Bevacizumab + capecitabine as maintenance therapy after initial bevacizumab + XELOX treatment in previously untreated patients with metastatic colorectal cancer: phase III 'Stop and Go' study results--a Turkish Oncology Group Trial. *Oncology*. 2013;85(6):328-335.

160. Yamada Y, Takahari D, Matsumoto H, et al. Leucovorin, fluorouracil, and oxaliplatin plus bevacizumab versus S-1 and oxaliplatin plus bevacizumab in patients with metastatic colorectal cancer (SOFT): an open-label, non-inferiority, randomised phase 3 trial. *The Lancet Oncology*. 2013;14(13):1278-1286.

161. Yamazaki K, Nagase M, Tamagawa H, et al. Randomized phase III study of bevacizumab plus FOLFIRI and bevacizumab plus mFOLFOX6 as first-line treatment for patients with metastatic colorectal cancer (WJOG4407G). *Annals of oncology : official journal of the European Society for Medical Oncology*. 2016;27(8):1539-1546.

162. Yasui H, Muro K, Shimada Y, et al. A phase 3 non-inferiority study of 5-FU/l-leucovorin/irinotecan (FOLFIRI) versus irinotecan/S-1 (IRIS) as second-line chemotherapy for metastatic colorectal cancer: updated results of the FIRIS study. *Journal of cancer research and clinical oncology*. 2015;141(1):153-160.

© 2022 Shen C et al. *JAMA Network Open.*
